# Microorganisms in Fermented Apple Beverages: Current Knowledge and Future Directions

**DOI:** 10.3390/microorganisms5030039

**Published:** 2017-07-25

**Authors:** Fabien J. Cousin, Rozenn Le Guellec, Margot Schlusselhuber, Marion Dalmasso, Jean-Marie Laplace, Marina Cretenet

**Affiliations:** Aliments Bioprocédés Toxicologie Environnements, Normandie Univ., UNICAEN, UNIROUEN, 14000 Caen, France; fabien.cousin@unicaen.fr (F.J.C.); rozenn.leguellec@unicaen.fr (R.L.G.); margot.schlusselhuber@unicaen.fr (M.S.); marion.dalmasso@unicaen.fr (M.D.); jean-marie.laplace@unicaen.fr (J.-M.L.)

**Keywords:** apple, cider, lactic acid bacteria, fermentation, organoleptic quality, safety improvement, microbial diversity

## Abstract

Production of fermented apple beverages is spread all around the world with specificities in each country. ‘French ciders’ refer to fermented apple juice mainly produced in the northwest of France and often associated with short periods of consumption. Research articles on this kind of product are scarce compared to wine, especially on phenomena associated with microbial activities. The wine fermentation microbiome and its dynamics, organoleptic improvement for healthy and pleasant products and development of starters are now widely studied. Even if both beverages seem close in terms of microbiome and process (with both alcoholic and malolactic fermentations), the inherent properties of the raw materials and different production and environmental parameters make research on the specificities of apple fermentation beverages worthwhile. This review summarizes current knowledge on the cider microbial ecosystem, associated activities and the influence of process parameters. In addition, available data on cider quality and safety is reviewed. Finally, we focus on the future role of lactic acid bacteria and yeasts in the development of even better or new beverages made from apples.

## 1. Introduction

Although styles of cider are extremely diverse and not easy to categorize, depending on the type of apple juices used and the degrees of sweetness, from extra dry to sweet, and alcohol content, ranging from 1.2–8% (*v*/*v*), cider can be defined as a fermented alcoholic beverage made from apple juice [1]. Cider production is encountered in more than 25 countries around the world in temperate regions where apple trees can flourish. The highest production is in Europe where the term cider refers strictly to fermented products [2,3]. Within Europe, the main cider-producing countries are England, Spain, France, Germany and Ireland, while smaller productions are found in Finland, Poland, Austria and Switzerland. The consumption of cider remains mainly European, accounting in 2016 for about 60% of world consumption compared with only 12% in North America [4]. There are several cider types, and traditional cider countries like Great Britain and France have their own specialties [5]. French cider tends to be sweeter than the sharper, drier cider of Great Britain, which has an alcohol content up to 8.5% (*v*/*v*). The fruity characteristics and aromas of French cider often are the result of ‘defecation’, in which pectins and other substances are separated from the juice. Then, the clear juice is raked off and fermented slowly and not to complete dryness [6]. In North America and Australia, the word ‘cider’ refers to the raw pressed unfermented apple juice, while ‘hard cider’ denotes a fermented product [7].

Cider is one of the oldest known beverages with a long and fascinating history. Historians broadly agree that apple trees existed along the Nile River Delta as early as 1300 BC [8], and a number of written documents citing alcoholic beverages made from apple and pear date back to ancient times, notably from Pliny, St. Augustin and Palladius [9]. By the beginning of the ninth century, cider drinking was well established in Europe, and a reference made by Charlemagne clearly confirms its popularity [10]. After the Norman Conquest of 1066, cider consumption became widespread in England, and orchards were established specifically to produce cider apples. In the first half of the twentieth century, cider was the second most consumed drink in France, behind wine, but ahead of beer [11]. Unfortunately, the damage caused to the Norman orchards during World War II together with the lack of public support resulted in a drastically reduced production, marking the decline of cider consumption in France. The current methods of cider production (quality of equipment, control assemblies and processes, stability, hygiene, neutralization of microorganisms, bottling, etc.) limit defects in the final product and make it possible to meet consumer requirements. Current cider producers use high quality standards, and ciders are elaborated under controlled conditions throughout the process.

**Figure 1:**
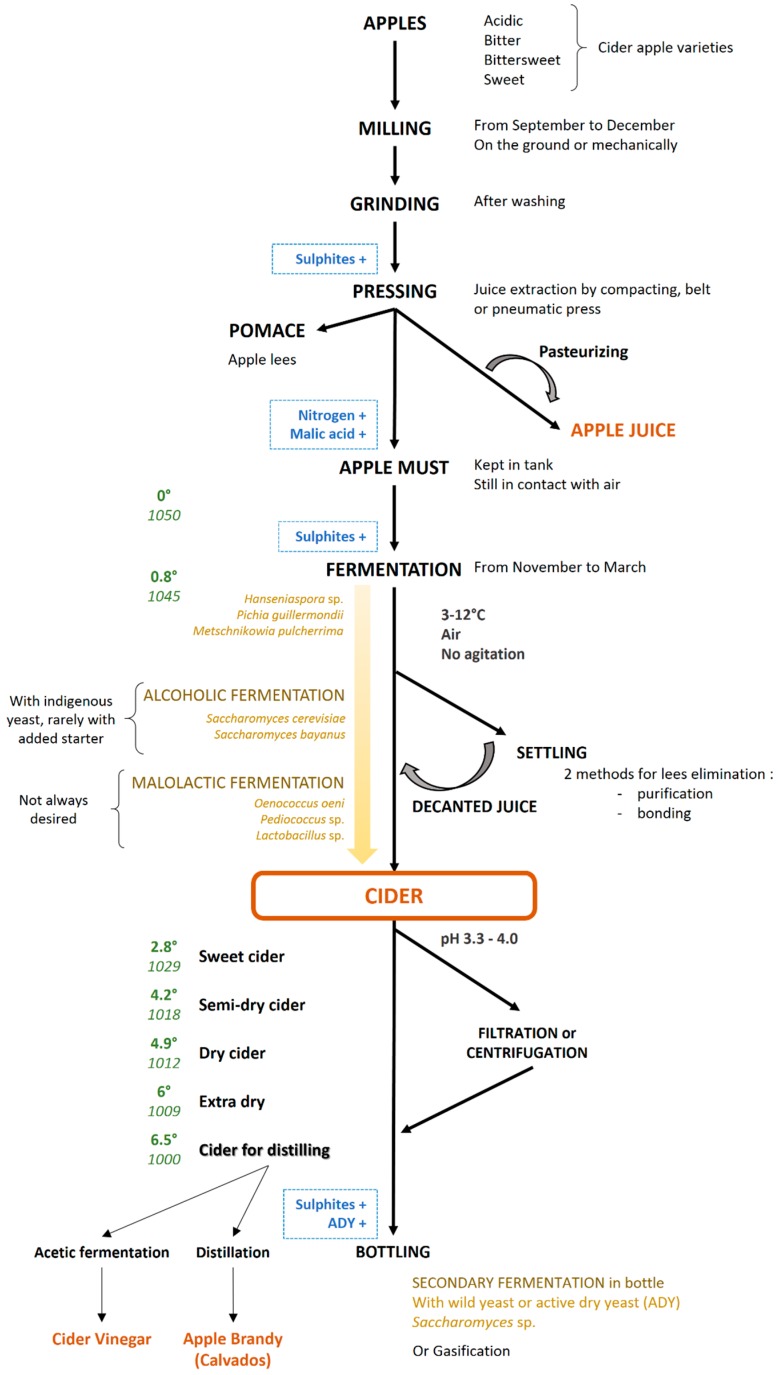
Cider-making process in France. Legend: +, optional addition; alcohol degree; density; ADY: active dry yeast.

In brief, the cider-making process (Figure 1) typically involves three main stages: apple crushing and pressing out the juice, followed by the most important stages of elaboration, fermentation. This includes classical alcoholic fermentation of sugars into ethanol performed by yeast strains and malolactic fermentation (MLF) processed by lactic acid bacteria (LAB) that can occur during the maturation. Although external sources of microorganisms may be added to the must in French traditional cider-making, alcoholic and malolactic fermentations are mainly performed by indigenous flora present on apples, on production equipment and in the cellar. Spontaneous fermentation begins within a few hours if the temperature of the must rises above 10 °C. This process is usually slow requiring at least 2–3 weeks for the main fermentation and several months for the maturation. Maturation takes place in wooden, polyester or stainless-steel casks at a controlled temperature of 3 °C–12 °C. The entire process can take from 1–6 months depending on the country. First, during alcoholic fermentation, sugars are converted mainly into ethanol and carbon dioxide by yeasts (mainly *Saccharomyces* sp.). The varietal choice and maturity of the fruits influence the sugar content of the starting must and, thus, the final ethanol level. Then, the malolactic fermentation involves the conversion of malic acid into lactic acid and carbon dioxide. Finally, the cider is bottled when its density is between 1009 and 1029 depending on the degree of sweetness desired (in France, typically extra dry, dry, half-dry and sweet). Active dry yeast (ADY) may be added in cider before bottling to obtain a naturally-carbonated beverage. The amount of residual sugar in the cider is essentially a consequence of the technological choice of the main alcoholic fermentation stoppage density and of the cider density at bottling. As shown in Figure 1, sulfites may be added at different stages of the process. Before fermentation, sulfites are added to control the natural microflora and to minimize oxidation of apple juice constituents. At bottling, sulfites are used to prevent oxidative changes and to inhibit secondary contamination [12]. After pressing, malic acid may also be added; this practice is a simple and effective way to change the acidity of the must.

Cider is a fermented beverage for which the recognition of ‘territoriality’ is important for its appreciation. The sensory profile of cider is significantly associated with microbial activities, and indigenous microorganisms may actively contribute to the expression of cider typicity. The microbial ecology of ciders is complex and includes several genera, species and strains of yeasts and bacteria [13,14]. During must production, fermentation and in the post-fermentative stage, apple juice or cider is susceptible to alteration by oxygen, enzymes, heat and/or microorganisms that can lead to a loss of nutritional and organoleptic qualities. With the increasing demand of consumers for nutritious, healthy and fresh-looking products with high organoleptic qualities, measures have been developed to prevent such alterations and to control the organoleptic characteristics of the product. 

This review aims at describing the role of microbial flora in the fermentation of apple juices, highlighting the links between ecological factors, yeasts and LAB diversities and the organoleptic properties of ciders. To date, even if ciders are safe products, research has focused mainly on the quality and safety of ciders through studies on the limitation of the development of spoilage and pathogenic microorganisms. This review will focus more on microbial quality referring to the overall effects of microbial activity, including growth, enzymatic activity and metabolic byproducts. Finally, a review of microbial diversity and the microbial contribution to the quality and safety of ciders will give us the opportunity to propose new perspectives for research on apple fermented beverages, especially through LAB activities.

## 2. Microbial Diversity: From Apple to Cider

Regarding the microbial ecosystem, next generation sequencing strategies bring many new whole ecosystem pictures, especially regarding non-cultivable bacteria [15,16]. Such studies are very rare in the cider research area, with only one paper revealing the microbiota of wine and organic apple cider submerged vinegar production [17]. Another study described the yeast biodiversity in must and during alcoholic fermentation [18]. There is a major lack of data on cider microbiota and its dynamics during the process. Current microbial research has moved into the genomic era with increasing amounts of data available, along with decreasing costs for sequencing, especially for LAB [19] and more specifically lactobacilli [20]. Better knowledge could be easily obtained by specific (meta-)genomic analysis of cider microbiomes.

### 2.1. Yeast and Mold Diversity 

Fungi (yeasts and molds) are naturally present on apples and can be found at each step of cider production. The presence of yeasts at the early stage of flower blossom has been described in various plants [21,22]. In flower nectar, yeast levels can reach densities up to 4 × 10^8^ cells/mL, and their frequency and abundance are directly correlated with the proportion of floral visits by bumble-bees, which thus appear as potential transmission vectors of yeasts from one flower to another in an orchard [21]. However, it seems that for the majority of plant nectars, the diversity of yeast communities is rather low [22]. On apple blossoms, yeasts have been isolated from both stigma and hypanthium surfaces, at frequencies similar to or greater than bacteria, particularly in hypanthia [23]. 

The apple surface is also a natural reservoir of fungi. In freshly-cut apples, fungi levels can range from 3.6–7.1 log CFU/g [24]. The dominant species identified in these cut apples were *Candida sake* and *Pichia fermentans*. Some of the fungi species on apples can be phytopathogenic species mainly included in the class Dothideomycetes, with about 95% of these in the order Capnodiales that causes damaging blemishes on apples [25,26]. A PCR-DGGE based-study of the microbiota of five varieties of Asturian apples used for the production of PDO (Protected Designation of Origin) ciders in Spain also identified *Exobasidium* sp., responsible for galls and leaf malformations, and *Mycosphaerellaceae* and *Dissoconiaceae* families, which produce sooty blotch and flyspeck on apples [27]. In this work, little variation in microbial diversity was found amongst the five apple varieties studied, without identifying the usual species associated with spontaneous fermentation. The authors conclude that the surface microbiota of the apples does not seem to be a determinant in the subsequent fermentation process. In contrast, another study showed that apples themselves can be the source of yeasts of technological interest [28]. This was the case with *Saccharomyces cerevisiae* yeasts, which could be found in high numbers on apples used for traditional Irish cider fermentations. In the same way, *Hanseniaspora* and *Brettanomyces/Dekkera* in ciders could be tracked back to the fruits.

The main yeasts found in cider are *Saccharomyces* yeasts. A study of unpasteurized ciders and cider musts obtained from different cider houses from northwestern regions of France reported 15 yeast species among 208 picked isolates [29]. The main species in this study was *Saccharomyces bayanus* accounting for 34.5% of the isolates, followed by *Saccharomyces cerevisiae*, *Lachancea cidri*, *Dekkera anomala* and *Hanseniaspora valbyensis* representing 16%, 15%, 10.5% and 6.5% of the isolates, respectively. The proportions of each of the 10 other species, i.e., *Candida oleophila*, *C. sake*, *C. stellate*, *C. tropicalis*, *H. uvarum*, *Kluyveromyces marxianus*, *Metschnikowia pulcherrima*, *Pichia delftensis*, *P. misumaiensis* and *P. nakasei*, never exceeded 3.5% of the total isolates. Yeast diversity was higher in cider musts than bottled ciders. Regarding the dominance of *S. bayanus*, the same observation was made in natural cider from Asturias (Spain) [30]. *Saccharomyces bayanus* was the predominant species from the beginning to the middle steps of the fermentation process, accounting for up to 41% of the picked isolates, whereas *S. cerevisiae* took over the process in the final stages of fermentation. *H. valbyensis* was always present at the end of fermentations regardless of the fermentation process used. The variations in the proportions of the different identified yeasts are connected to the occurrence of a sequential succession of yeast species throughout the cider-making process. Morrissey et al. thus identified three phases in the cider process based on the dominant yeast species present [28]. The first phase, which they called ‘the fruit yeast’ phase, is dominated by *Hanseniaspora uvarum*/*Kloeckera apiculata* yeasts, along with a few *S. cerevisiae* yeasts [14,28]. The second phase, or ‘fermentation phase’ where the alcoholic fermentation occurs, is characterized by the replacement of oxidative or slightly fermentative non-*Saccharomyces* yeasts by the strong fermenting *Saccharomyces* yeasts, such as *S. bayanus* and *S. cerevisiae*. The last ‘maturation phase’ is dominated by *Brettanomyces/Dekkera* yeasts. The yeast population fluctuates from one year of production to the next [31]. This is visible in the variations in the proportions of the main yeast species constituting a resident mycoflora throughout cider cellars and by the intermittent apparition of some species constituting a ‘transitory mycoflora’. 

### 2.2. Bacterial Diversity

Bacteria are present from the apple flowers to the final product (Table 1). In 2013, Shade et al. studied the apple flower microbiome by pyrosequencing and described the presence of diversified bacterial communities evolving differently from the bud to the fruit [32]. This study highlighted that apple flowers carry bacteria that will be involved in the process of cider or vinegar making (mainly *Lactobacillaceae* and *Acetobacteraceae* families, respectively). Surprisingly, bacteria from *Deinococcus-Thermus* phylum were found in abundance. This phylum was not known to be related to fruit crop. *Enterobacteriaceae*, commonly isolated on apple fruits, were present at every stage of the flower maturation.

In 2015, Graça et al. detected principally mesophilic and psychrotrophic microorganisms on fresh cut apple while coliforms and LAB were isolated on apple flowers [24]. Focusing on cider apples, Alonso et al. used PCR-DGGE to study the native microbiota of five apple varieties commonly used in the Asturian cider-making process. Predictably, *Enterobacteriaceae* were present due to the ubiquity in nature of this genus, but bacterial species usually associated with spontaneous fermentation were not [27]. The apple surface microbiota may not be a determinant in the fermentation process. The microbiota of apple cider is strongly influenced by other factors such as harvest techniques, quality sorting and storage. In 2004, Keller et al. brought to light the influence of picking techniques on the microbiota [42]. Cider apples picked from the ground after their fall bring more bacterial diversity than those tree harvested. After grinding, no difference between bacteria counts were found, whether they were stored or not. However, significant differences in bacterial counts between apple varieties were identified.

Bacterial starters do not exist yet in cider; thus, Sanchez et al. investigated LAB prevalence during the malolactic fermentation in Asturian cider cellars in order to find the most efficient fermentative strains [33]. They mostly isolated strains of *Lactobacillus brevis* and *Oenococcus oeni.* This last species is already known to be very tolerant to low pH and to the presence of alcohol [38]. According to a fermentation capacity evaluation of the selected strains, *O. oeni* strains were the most efficient. Salih et al. also highlighted the importance of *O. oeni* during the malolactic fermentation, and the presence of *Lactobacillus brevis* in some of the ciders tested [37]. Different behaviors of the LAB flora depend on the kind of apples used for cider-making (sweet cider apples, sweet dessert apples, bitter cider apples). The influence of the geographical origins of the indigenous cider LAB was determined by Sanchez et al. using the RAPD (Random Amplification of polymorphic DNA) technique on *O. oeni* strains. Five distinct groups, specific to only one producing area, were identified and had an identical RAPD profile. This significant result brought to light the link between *O. oeni* strains and their geographical origin [33]. A recent study focusing on the biogeography of *O. oeni* confirmed the importance of genetic adaptation of this species in cider and also highlighted that *O. oeni* from wine or from cider were genetically different [38]. The first genome of *O. oeni* has been sequenced and annotated in 2005 [43]. Many studies have investigated the genome of this bacterium and have shown that *O. oeni* strains from wine or cider present a different genomic content [44,45,46]. A recent study of Sternes et al. analyzed the pan-genome of *O. oeni* with 191 strains, of which only four have been isolated from cider [46]. They showed again that three out of four of the cider isolates cluster closely together. The presence of neighboring wine-derived strains suggests that information from additional strains isolated from cider is required before any conclusion regarding the possibility of a cider-specific subset of *O. oeni* can be reached. The other source of genomic data from LAB isolated from cider is related to their technological or probiotic potential [47,48].

*Lactobacillus* sp. and *Oenococcus* sp. are the most common LAB identified in apple juice byproducts. In apple cider vinegar, which is the result of acetic fermentation, both of them were detected, even if acetic acid bacteria, such as *Acetobacter* sp., *Komagataeibacter* sp. or *Gluconobacter* sp., were the most abundant [17]. In 2010, Sanchez et al. studied the LAB diversity during malolactic fermentation in an industrial cider [13]. Using molecular tools, such as 16S rRNA gene sequencing, *Lactobacillus collinoides*, *O. oeni*, *Pediococcus parvulus* and, with minor content, bacteria like *L. casei* or *P. ethanolidurans* were identified. Acetic acid bacteria are necessary for vinegar production, but can ruin cider production. In contrast, LAB are essential in malolactic conversion during cider production, but some can damage the product by producing spoilage compounds. 

### 2.3. Factors Influencing Microbial Diversity

Variations in the microbial ecosystem of ciders are associated with several factors, from the orchards to the final product. First, microbial diversity is determined by the growing conditions of the fruits such as the apple varieties, the climate and the production process. The cultivation practices have an impact on the fruit microbial composition in terms of abundance and diversity. Organic and conventional apple bacterial communities were shown to be significantly different [49,50]. The organic apple phyllosphere displayed higher numbers of bacteria than the conventional apple phyllosphere. A comparison of integrated and organic growing systems for Golden Delicious apple production also revealed significantly higher frequencies of filamentous fungi, greater abundance of total fungi and of taxon diversity in organic apples than in integrated apples [51]. The crop management methods thus influence the microbial communities associated with the surface of apple fruits used for cider production. The apple variety also has an influence on the microbial composition of the fruits. Keller et al. showed that significant differences exist in total aerobic bacterial and fungal populations among apple varieties in relation to their pH, Brix and titratable acidity [42]. The apple varieties with the lowest titratable acidity, highest pH and highest Brix have the highest microbial concentrations (≥2.5 log CFU/g). The method of harvesting also plays a role in microbial diversity. Microbial populations on apples, in pomace and in cider are higher when apples are harvested off the ground rather than tree-picked. In the final cider, the average aerobic plate counts for all pooled varieties tested in the ground-harvested group was 4.89 log CFU/g compared with 2.88 log CFU/g for the fresh tree-picked group [42]. 

After fruit harvesting, the cider process modulates the microbial composition of ciders. The culling of apples result in ciders with higher microbial numbers than those made from unculled apples [42]. A strong link exists between the temperature profile of the cider fermentations and the yeast population dynamics of the predominant yeast species, present within the fermentations [28]. Another piece of research also showed that the musts obtained by pneumatic pressing were dominated by non-*Saccharomyces* yeasts (*Hanseniaspora* genus and *Metschnikowia pulcherrima*), whereas in the apple juices obtained by traditional pressing, *Saccharomyces* together with non-*Saccharomyces* were always present [30].

Cider processing facilities and cellars walls, floors and surfaces also constitute reservoirs of bacteria and fungi throughout the cider process. For example, one source of *S. cerevisiae* yeasts appears to be the process utensils, the press house and the vat-house, in which this resident flora can be found even six months after the last pressing [28].

Even if microbial reservoirs are broad, the microbial diversity and microflora successions also greatly depend on the aptitudes of the bacterial and fungal strains to resist or adapt to the process conditions such as depletion in oxygen levels, sulfites presence, CO_2_ and alcohol productions and essential nutrients’ availability. It also depends on the differences in their specific growth rates, in their sugar uptake capabilities, on inter-specific competition, cell death, flocculation and/or natural sedimentation characteristics [52].

## 3. Microbial Contribution to Cider Organoleptic Quality

### 3.1. Yeast Contribution 

During alcoholic fermentation, many byproducts such as esters, higher alcohols and phenolic compounds are produced as secondary metabolites. Esters provide mainly fruity and floral notes; higher alcohols provide ‘background flavors’; whereas the phenolic compounds can generate interesting or unpleasant aromatic notes. Esters are the main volatile compounds in cider behind ethanol [53]. They are characterized by a high presence of ethyl acetate, which alone can represent up to 90% of the total esters [54,55]. The amount of acetates produced by yeasts seems to be strongly related to the nature of the strains leading to alcoholic fermentation: *Saccharomyces* sp. produce fewer acetate amounts than non-*Saccharomyces* yeasts. Comparing the potential of *H. valbyensis* and *S. cerevisiae* to produce volatile compounds, Xu et al. [55] showed that *H. valbyensis* yielded higher concentrations of ethyl acetate and 2-phenylethyl acetate, while *S. cerevisiae* kept more free (non-esterified) isoamyl alcohol and isobutanol. A small variation in the ester concentration of ciders may have significant consequences on their final sensory quality [56]. Most of the esters are responsible for the fruity characteristics of ciders. However, an excessive amount of ethyl acetate may lead to an unpleasant smell of solvent.

Higher alcohols are directly derived from the metabolism of yeasts. They are synthesized during fermentation from oxo-acids originating in amino acids and sugar metabolism [57]. In ciders, they are mostly represented by isopentanols (2- and 3-methylbutanol) followed by isobutanol, propanol, butanol or hexanol [58]. Although they constitute a relatively low amount of the total substances, higher alcohols may greatly influence sensory characteristics. Rapp and Mandery [59] found the total higher alcohols in wine to be in the range 80 ± 540 mg/mL ; concentrations up to 300 mg/L contribute to pleasant flavor, but concentrations above 400 mg/mL provoke unpleasant flavor and harsh taste. Some higher alcohols, particularly iso-amyl alcohol, contribute to unpleasant flavor [60], although a positive correlation has been reported between n-butanol and the aroma quality of apple juice [61].

The third class of secondary products, i.e., the phenolic compounds, also have important effects on the sensory properties of apple ciders by either their content or their profile. These compounds derived from raw material have an impact mainly on color, bitterness, and astringency [62]. High molecular weight procyanidins in ciders are known to contribute to astringency, whereas the smaller compounds contribute to bitter taste [63,64,65]. Simultaneously, they influence the sweetness and sourness, thus further highlighting their importance in overall flavor development [64]. In addition to the non-volatile phenolic compounds, the volatile phenolics mainly formed by enzymatic decarboxylation during fermentation contribute to aroma [66].

It has been reported that during the early stages of fermentation, excess growth of the apiculated yeast *Kloeckera* can generate high levels of esters and volatile acids [67]. In wine, the aromatic profile is negatively influenced by the yeast *Brettanomyces*/*Dekkera* and is characterized by mousy, medicinal, wet wool, burnt plastic or horse sweat smells [68]. Buron et al. have shown that *Brettanomyces*/*Dekkera* cider strains were able to produce 4-ethylcatechol, 4-ethylphenol and 4-ethylguaiacol from caffeic, *p*-coumaric and ferulic acids, respectively [69]. These volatile phenols are associated with organoleptic defects. In contrast, in some beers, this yeast is considered essential and beneficial [70]. In wine- and cider-making on an industrial scale, the control of *Brettanomyces*/*Dekkera* is usually achieved through the addition of sulfur dioxide (SO_2_) to the fermentation medium [71]. In cider-making, the concentration of SO_2_ is in the range of 50–150 mg/mL at pH 3.0–3.8, not exceeding 200 mg/mL in total [72]. However, some strains of *Brettanomyces*/*Dekkera* are naturally resistant to SO_2_, and elimination of this yeast by physical treatments (filtration) has a limited efficiency (due to the cell size of this yeast) and does not prevent subsequent recontamination. 

### 3.2. Bacterial Contribution

Transformation of malic acid, lowering total acidity, is the major organoleptic change induced by LAB. During MLF, the strong green taste of malic acid is replaced by the less aggressive taste of lactic acid [73]. However, LAB are also responsible for other changes in aromas increasing flavor complexity, involving changes of fruity, flowery and nutty flavors, as well as the reduction of vegetative/herbaceous aromas by reduction of acetaldehyde metabolism [74,75,76].

*Lactobacillus*, *Leuconostoc*, *Oenococcus* and *Pediococcus* are genera of special interest as they are able to survive cider environments (low pH, high ethanol content and low nutrients). Research focuses on the contribution of *O. oeni*, but other genera, particularly *Lactobacillus* species, should not be underestimated [77]. In wine, it is well known that some varietal aromas revealed during alcoholic fermentation by yeast disappear or change after malolactic fermentation [73]. For example, the concentration of some esters can be either increased or decreased by MLF, according to the type of bacterial strain used [78]. Apart from esters, aroma compounds such as higher alcohols, fatty acids, lactones and sulfur and nitrogen compounds can be produced by LAB [77]. 

LAB contribution to aromatic profiles of ciders has been explored less than it has been in wine. A few studies are available, principally linked on the use of *O. oeni* strains as starters rather than studying LAB metabolism in the cider environment. In wine, LAB contribution is focused on citric acid metabolism that induces the production of compounds linked to buttery descriptors: diacetyl, 2,3-butanediol and acetoin [79]. Together with acetonic compounds, citric acid degradation involves the production of acetic acid that can significantly modify the aromatic profile. Citric acid metabolism with the production of diacetyl cannot be responsible for the whole panel of flavor modifications, and the mechanisms should be further studied. 

Along with the favorable sensory changes that can occur during cider elaboration, LAB can be also responsible for undesirable reactions. The frequent cider alteration known as ‘piqûre acroléique’ is mainly caused by a heterofermentative LAB commonly encountered in cider, *Lactobacillus collinoides* [80,81]. In apple-derived products, this alteration results from glycerol degradation to 3-hydroxypropionaldehyde (3-HPA) under the action of *L. collinoides* via the diol-dehydratase enzyme. In addition to *L. collinoides*, some other cider species, like *L. hilgardii* [34] or *L. diolivorans* [82], are able to produce 3-HPA. Glycerol is one of the major products of yeasts metabolism during cider alcoholic fermentation and is important for the sensorial quality of fermented beverages. Due to its high instability, during the distillation process, the 3-HPA is transformed by dehydration [83] in acrolein, a lachrymatory chemical generating a peppery flavor, which can spoil the product, giving a bitter taste [84,85].

One major spoilage microorganism is the Gram-negative, facultative anaerobic bacterium *Zymomonas mobilis* isolated from various alcoholic beverages, including ciders, beers and perries. *Z. mobilis* is a remarkable bacterium and a very promising microorganism for industrial ethanol production because its catabolism follows the Entner–Doudoroff pathway, thus giving a near-theoretical yield of ethanol from glucose, fructose and sucrose, the only carbon and energy sources that support its growth [40]. As a cider spoilage microorganism, growth of *Z. mobilis* is correlated with the production of large quantities of acetaldehyde along with an almost explosive production of gas and a marked turbidity of the product, an alteration known as ‘framboisé’ in French ciders or ‘cider-sickness’ in English ciders [41,86]. Associated with these symptoms is a marked change in the flavor of the beverage, the original fruity character being lost or hidden by a strong and characteristic taste, reminiscent of raspberry. Malolactic fermentation (MLF) is considered to enhance the risk of ‘framboisé’, and Bauduin et al. [41] have shown that the relationship between MLF and ‘framboisé’ is mainly associated with the increase of pH correlated with the conversion of malic acid to lactic acid rather than with nutritional factors produced by LAB. In fact, the amount of residual nitrogen in cider appears to be the main factor controlling the growth of *Z. mobilis*, and thus, a solution for the prevention of this alteration consists of reducing the amount of residual nitrogen as soon as possible [41].

Therefore, a greater knowledge of cider LAB flora and their metabolisms in a cider environment could provide laboratory and practical cellar tools for a better control of cider quality.

## 4. Safety Assessment of Fermented Apple Beverages

Fermented foods and beverages are known to be safer than unfermented counterparts. The improved food safety arising from fermentation is largely due to LAB, a predominant group of organisms in most fermented foods and beverages. Occasionally, bacterial pathogens such as *Salmonella* spp., *Escherichia coli* and *Staphylococcus aureus*, originating from orchard soil, farm and processing equipment or human sources, may occur in apple juice. However, both apple juice and fermented cider contain organic acids, mainly malic acid (≅5 g/L) in apple juice and lactic acid (3–4 g/L) in fermented cider, generating acidity (pH level ranging from 3.0–3.5 and 3.3–4.0, respectively) that usually prevents the growth of these pathogens, which can survive for only a few hours. The growth and metabolism of LAB usually inhibit the growth of normal spoilage flora of the matrix and of any bacterial pathogens that it may contain. Therefore, apple cider is traditionally not regarded as a potentially hazardous food [87]. However, the monitoring of food-borne hazards in cider such as the pathogenic bacteria *E. coli*, protozoan *Cryptosporidium*, biogenic amines or mycotoxins still requires vigilance on the part of cider producers.

### 4.1. Biogenic Amines

Biogenic amines (BA) are low molecular weight organic bases with an aliphatic, aromatic or heterocyclic structure frequently occurring in foods and beverages involving fermentation or the ripening process. The formation of these molecules is achieved through the removal of the alpha carboxyl group from amino acids [88]. The most abundant BA found in foods are histamine, tyramine, putrescine, cadaverine and phenyl ethylamine. In fermented beverages, such as beer, wine and cider, production is influenced by microorganisms present [88,89], environmental factors such as pH, ethanol [90,91], sulfur anhydride level [92], raw material quality and fermentation, as well as technological conditions [90,93]. Consumption of food containing high level of BAs can induce adverse reactions such as headache, hyper- or hypo-tension and rashes. Such disorders may become serious especially for consumers whose detoxification system is impaired either by genetic disorders or medical treatments [89]. Histamine and tyramine are considered as most toxic and particularly relevant for food safety, while putrescine and cadaverine are known to potentiate these effects [94]. 

In cider, as microbiological stabilization is not performed after MLF, indigenous heterofermentative LAB constitute the predominant flora capable of promoting the production of BAs [36,39,95]. As shown in Table 2, among LAB, *Oenococcus* and *Lactobacillus* were found to be the most representative genera of BA producers in cider. 

Studies conducted on commercial cider from Spain and France revealed the presence of BA in almost 90% of the analyzed products with a higher prevalence of tyramine, histamine, putrescine and cadaverine among other amines [89,95]. Nevertheless, BA content of cider seems to be lower than that detected in other fermented foods and beverages [88]. Some differences in amount and composition were also found between French and Spanish samples. More precisely, cadaverine and putrescine were detected at a maximal concentration of 34 mg/L in 20% and 57% of Spanish cider samples, respectively, while only in trace amounts in only a third of French cider samples (1 mg/L). Tyramine was the most frequently detected BA in French samples (present in 70% of samples in concentrations below 14 mg/L). Histamine was detected at relatively low levels in both French and Spanish samples (26% of total samples, below 16 mg/L) [89,95,96]. As mentioned by Ladero et al., the characteristics of the apple variety used and/or the different elaboration processes, as well as possible microbiota differences could explain the differences [89]. The global amount and profile of BA produced do not appear to be driven by cider-making steps and types of press [95]. Therefore, some strategies have been proposed to decrease the formation of BA, such as (a) reducing amino acid precursor levels (generally decreasing with fruit ripening), (b) limiting the growth of spoilage bacteria, (c) inoculating starter cultures without amino acid decarboxylase and (d) inoculating biogenic amine-degrading microorganisms [94,97].

### 4.2. Mycotoxins

Mycotoxins are secondary metabolites of filamentous fungi mainly triggered by *Aspergillus*, *Fusarium* and *Penicillium* genera [98]. In apple and apple-derived products, patulin represents the most relevant mycotoxin. The toxin is an unsaturated heterocyclic lactone toxin produced by a wide range of mold species [99]. Among these species, *P. expansum,* as the main pre-harvest and post-harvest contaminant in pomaceous fruits (apples and pears), is considered as the major source of patulin in these fruits [100]. The level in food and beverages is regulated in Europe by the European Commission and in the United States by the U.S. Food and Drug Administration (FDA) to a maximum acceptable concentration of 50 µg/L for fruit juices and derived products (including cider) [101]. Indeed, Michigan apple cider mills were analyzed for patulin concentration. The mycotoxin was detected in almost 20% of cider mill samples with 2% of samples having concentration higher than 50 µg/L [102]. Temperature, activity of water (a_w_) and pH were found to influence *P. expansum* growth, as well as patulin production. The fungi is able to produce the toxin around 16 °C. *P. expansum* can produce the mycotoxin only at an a_w_ of 0.99, which is the approximate a_w_ of fresh fruits. Finally, patulin production was found to be optimum at pH 4 [103]. Apple contains natural acids (citric and malic acids) that lead to the reaching of these optimal conditions (pH of fruit varies from <2.5–5) [104]. It is commonly admitted that the toxin is generally unstable during fermentation, so that products such as cider are usually free of patulin. In a recent study, the level of patulin in contaminated musts was shown to have decreased six-fold after two days of fermentation [105]. Reports of patulin in cider are likely due to the adjunction of apple juice to produce ‘sweet cider’ or low-fermented cider [102]. 

### 4.3. Pathogens

As previously mentioned, unpasteurized apple cider is historically considered to be a safe product, free of microbial pathogens due to its acidic level and to the fermentation process. However, some bacterial and parasitic pathogens can survive and may remain infectious [106]. To date, enterohemorrhagic *E. coli* (EHEC) serotype O157:H7, as well as the protozoan *Cryptosporidium parvum* have been linked to several outbreaks since the 1980s due to apple cider consumption [106]. Nevertheless, it is important to notice that such outbreaks only occurred in North America and mainly in unfermented apple ciders [106]. European apple cider has never been implicated in any outbreaks of this kind due to alcoholic fermentation process byproduct, ethanol, which is toxic for most potential pathogens in cider [107]. EHEC O157:H7 is known to have a fecal origin and may contaminate apples, juice and cider directly from animal/human feces or by indirect contact (equipment, contaminated water, etc.) [108]. *Cryptosporidium* spp. is an intracellular parasite with an infectious stage known as oocysts. Oral transmission of the parasite is facilitated by the ability of oocysts to survive for weeks to months in the environment. The study of contamination sources in unpasteurized apple cider revealed that the parasite is found in washed apples, water, fresh and finished cider [108]. Kniel et al. studied the potential of malic acid, as well as hydrogen peroxide to reduce the infectivity of *C. parvum* in apple cider. Interestingly, infectivity was completely inhibited by incubation of oocysts in apple cider plus 0.025% H_2_O_2_ and inhibited (up to 88%) by the addition of 5% malic acid [109].

## 5. Functional Improvement of Apple Fermented Beverages

### 5.1. Control of the Microbial Ecosystem to Improve or Modulate Cider Quality

#### 5.1.1. Rational Design of Starter Cultures

The selection of appropriate starter strains is key in the control of the cider fermentation process and characteristics of the final beverage. Microbial starters, especially *O. oeni*, are less used in cider production than in wine production, but their role might be crucial for the quality of the final product [110]. This leads to many studies focusing on the selection of microbial starters [33,111], their improvement [112,113] and the use of other LAB than *O. oeni* [114]. The use of isolated strains of *S. cerevisiae* is an interesting strategy for maintaining the quality and reproducibility of fermented beverages. This is especially true for the Champenoise method, typical of Asturian PDO ciders, and based on a secondary fermentation in bottle. The screening and the selection of local yeast strains is believed to be more effective than using commercial starters, as these endemic strains are potentially better acclimated to the environmental conditions than industrial starters [115]. These authors thus proposed a methodology for the rapid screening and selection of autochthonous yeast strains based on their oenological and technological properties. The ciders obtained with the selected yeast strains were scored as good after sensory analysis. The choice in species driving the fermentation is important for technological purposes and also for the aroma profile development in cider. For example, the presence of *Hanseniaspora* sp. yeast strains during apple fermentation results in the production of considerable amounts of esters and alcohols, contributing to fruity sensory notes, compared with apple musts fermented only with *Saccharomyces* sp. yeasts, which provide rather neutral sensory notes [116]. The fermentation performance has also been improved by the use of a hybrid strain between *S. eubayanus* and *S. cerevisiae* [111]. Recently, in order to control the proliferation of *Brettanomyces*/*Dekkera* in wine, Ngwekazi et al. [71] have identified and characterized killer toxins secreted by non-*Saccharomyces* yeasts related to wine. Their results, although preliminary, show that killer toxins have a high potential to control the population of large numbers of *Brettanomyces*/*Dekkera* strains. These results are especially encouraging, as none of the killer toxins characterized inhibit the fermentative yeast *Saccharomyces*.

Another characteristic to be considered for the bacterial strains used in the cider fermentation process is their aptitude in resisting bacteriophages (phages). The presence of lytic or lysogenic phages of *Oenococcus* has previously been described in wine [117]. Recently, Constanti et al. characterized *O. oeni* bacteriophages and the related implications for malolactic fermentation in wine [118]. They reported that pH and ethanol affect the lytic activity of *Oenococcus* phages, especially when wine alcohol content is low. The presence of phages in cider has not yet been investigated. It is thus a point of interest that should be examined, as anti-*Oenococcus* phages could be at the origin of cider fermentation problems by disturbing the malolactic fermentation driven by phage-sensitive *Oenococcus* strains. Still, phages could be used as antimicrobial agents against spoilage and pathogenic bacteria in ciders and therefore help in controlling the safety and quality of these fermented beverages. Phage therapy in the food industry has been extensively studied [119]. However, phage applications to apple fermented beverages are scarce. One main obstacle to their efficient antimicrobial action would be their potential sensitivity to acidity found in apple products [120].

#### 5.1.2. Control of the Fermentation Process Parameters

Spoilage and pathogenic flora in apple juice and by extension in apple fermented beverage can be reduced by physical methods, which have already been reviewed [121] and will not be further discussed. The control of the fermentation process parameters such as the time of inoculation of mixed cultures, the temperature of fermentation steps in the process or prefermentative steps is a key element in the modulation of the cider ecosystems throughout processing and thus of the quality of the final product. Aroma production during cider fermentation is greatly dependent on the yeast species in the presence and their sequential succession throughout the process. A study of the co-culture of *Wickerhamomyces anomalus* and *S. cerevisiae* showed that the association could help improve the quality and add complexity to the cider [122]. Controlling the strain association parameters during the fermentation process, i.e., the inoculation time and the sequential or simultaneous mixed cultures, is crucial for the optimization of the desired kind of cider. In the same way, the fermentation of a vegetable juice using a mixed culture of *S. cerevisiae* and *L. plantarum* resulting in an enhancement of the nutritional content of the final beverage [123] emphasizes the feasibility of the chosen co-fermentation by the selection of the right microbial associations for designing new functional apple fermented beverages.

Yeast metabolism is greatly dependent on the temperatures applied during the fermentation process. Peng et al. [124] have shown that variations of the fermentation temperature have a direct influence on the aromatic profile of the final cider. In their study, the ciders fermented at 20 °C seemed to result in the best acceptance by the consumer and displayed the highest aromatic characteristics. This is probably due to modifications in the microbial metabolism that result in variations in the production of esters, volatile compounds and alcohols according to the fermentation temperatures. Variations in the fermentation temperature are nonetheless to be considered with care, as the increase of temperature can also lead to the formation of undesired compounds by the expression of unsuitable microbial metabolic pathways [124,125]. In the same way, fruit processing, pectinolytic enzyme application, cell yeast immobilization on alginate and the type of fermentation have all a significant influence on the antioxidant capacity, polyphenol profile and volatile composition of ciders [126]. Prefermentative treatments such as pulp fermentation induced the formation of higher amounts of ethanol, procyanidins B2 and C1, epicatechin and catechin and resulted in a higher antioxidant activity than in non-pulp fermented ciders. Cell immobilization positively affected the ethanol content, but decreased the antioxidant activity of ciders. Ciders obtained with spontaneous fermentation contained more esters and methanol compared to inoculated ciders [126]. The MLF is also a bottle-neck in the cider production process, and one area of research is looking for strategies to control/improve this natural phenomenon [127]. Such process parameters can thus be used as levers to modulate the quality of the final product.

#### 5.1.3. Control of Cider Quality by LAB

Microbial quality is obviously microorganism dependent and is highly affected by chemical, physical and biological factors pertaining to the environment. Maintaining microbiological quality and the maximum sensory and nutritional quality of fermented beverages requires a combination of antimicrobial hurdles in order to limit the growth of undesired microorganisms. By producing organic acids as a fermentation metabolite, antimicrobial peptides and hydrogen peroxide, LAB strains may contribute to improving the quality of apple ciders. Bacteriocins are generated from bacteria and, usually, are inhibitory towards phylogenetically-related species. There are only a few reports about the inhibitory activity of bacteriocins against yeasts [128,129,130]. To our knowledge, the effectiveness of bacteriocins from LAB for controlling the growth of undesirable yeasts in cider or wine has never been studied, although bacteriocins produced by LAB have received considerable attention over the years for their possible use as biopreservatives in food, to reduce the use of chemical preservatives. It could therefore be interesting to screen new bacteriocins from LAB isolated from fermented beverages.

Bacteriocins could also be effective against spoilage bacteria. *L. collinoides* exhibits natural resistance to conditions encountered during the fermentative process [131]. In order to avoid this alteration, the bacteriocin enterocin AS-48, a broad-spectrum antimicrobial peptide produced by *Enterococcus faecalis* [132], was tested against two 3-HPA-producing *L. collinoides* strains causing apple cider spoilage. The two *L. collinoides* strains tested were rapidly inactivated by low concentrations of enterocin AS-48 in fresh apple juice (2.5 µg/mL) and also in Basque cider (2.5–5 µg/mL) [133]. Another classical disorder, which does not affect flavor, is known as ‘ropiness’. This microbiological problem arises when certain bacteria synthesize exopolysaccharides (EPS), thus increasing the viscosity of the cider [134]. The EPS show a large variation in composition, molecular mass and structure and, once secreted into the medium, play an important role in the rheology and texture of fermented beverages, enhancing naturally the texture and viscosity [135]. As a consequence of the increase of viscosity, the cider flows like oil; hence the term ‘ropiness’. In addition to being a biothickener, prebiotic effects of several EPS have been demonstrated [136]; however, despite these interesting properties, a high level of EPS production in cider is unwanted, as it is prejudicial to the organoleptic quality of the product. Although ropiness is mainly caused by some strains of LAB [137,138,139], it has been shown that one strain belonging to the *Bacillus* genus can be responsible for this alteration [140]. Among the alternative methods suggested to avoid this alteration in beverages, Grande et al. [141] have tested the efficacy of the *E. faecalis* enterocin AS-48 against a slime-producing *B. licheniformis* strain in apple cider. Their results show that enterocin AS-48 is also active against the EPS-producing strain either in culture medium or in apple cider, suggesting a possible use of this enterocin to prevent ropiness. These results are of great interest for the development of tools allowing for the control of undesired bacteria in fermented apple cider.

Thanks to amino oxidase enzymatic activity, some species of LAB appear to be of great interest for the potential control of BA-related health risk. Hitherto, many studies have been conducted with the purpose to identify LAB isolated from fermented foods with BA degrading capability, but only a few concern fermented beverages. To our knowledge, no study has been conducted on LAB isolated from cider. A collection of 85 LAB isolated from wines, must and lees was screened for their ability to degrade histamine, tyramine and/or putrescine. Twenty-five percent of the LAB were able to degrade histamine, 18% tyramine and 18% putrescine. The strains with highest activity belonged to *Lactobacillus* and *Pediococcus* groups, and most of them were able to degrade simultaneously at least two BAs [108]. In the future, it might be of interest to screen the potential of cider-associated LAB to reduce potential BA level in this beverage.

Large numbers of studies attribute antifungal activity to LAB strains thanks to the production of various organic acids (such as lactic, acetic, caproic, formic, propionic, phenyl lactic and butyric), fatty acids and peptides [142,143]. LAB may represent interesting biological control agents in apple fermented beverages by means other than bacteriocin. The detoxification of patulin through binding to bacterial surface proteins is an example [144,145]. Recently, Zoghi et al. identified two probiotic strains of *L. acidophilus* and *L. plantarum* able to catch the toxin through their surface layer proteins (fructooligosaccharide content). In the best conditions and after six weeks of refrigerated storage, more than 90% of initial patulin were removed from apple juice with no significant difference in organoleptic properties [144]. 

### 5.2. Health Benefits of Apple Fermented Beverages

Fermented beverages and especially non-dairy probiotic beverages are believed to be the next functional foods for probiotic delivery. Likely candidates are chilled fruit juices or fermented vegetable juices [146]. For the consumer, they present the advantages of lacking dairy allergens such as lactose, containing low cholesterol and having a vegan-friendly status [147]. The health benefits of fermented beverages have been described. The improvement of gastrointestinal health associated with the microbial content of fermented beverage is thought to be responsible for perceived health outcomes. Evidence of the direct or indirect action of the beverage microbiota on gastrointestinal health have been given over the years, even if the mechanisms involved are still unclear for the most part [148]. The health benefits of apple beverages have been the subject of much scrutiny, for many years. For example, apple beverages, including cider, have been shown to have anti-viral properties [149]. Some apple juices are already used as vectors of probiotic lactobacilli strains [150,151]. Several traditional cereal and vegetal fermented beverages are the source of probiotic bacteria [152]. Apple fermented beverages can therefore be sources and vectors of probiotics. Spent cider yeast, a by-product of the fermentation process, was used as a dietary supplementation in a piglet model. This supplementation proved to enhance gut functions and to reduce *Salmonella* and *Escherichia* carriage in porcine gut [153]. Some probiotic potential has also been demonstrated for lactobacilli [48] or pediococci [47,154]. A probiotic beverage from apple fermented with *L. casei* has recently been developed for human consumption [151]. 

A recent review detailed the role of LAB as an efficient cell factory for the production of functional biomolecules and food ingredients to enhance the quality of cereal-based beverages [155]. These LAB assets could be transposed to apple fermented beverages. They encompass the LAB-mediated inhibition of spoilage or pathogenic microorganisms through antibacterial compound production, the reduction of potential antinutritive factors, the amelioration of the apple fermented beverage nutritional value, the LAB aroma and flavor compound production, the production of EPS related to texture development, organoleptic changes and the prebiotic nature of those beverages, the production of nutraceutical compounds and anti-allergenic biomolecules. Some LAB, isolated from wine or cider, also showed potential intrinsic (without grape/apple matrix) health benefits [156]. For example, *O. oeni* can harbor anti-inflammatory potential [157] or produce EPS [158]. This EPS production could even help with the industrial production of food products containing lyophilized *O. oeni* strains [158]. The EPS production by LAB could also be related to industrial perspectives such as viscosity and mouth feel enhancement properties [159].

The development of functional apple fermented beverages is promising. For example, strategies combining apple juice and a novel whey-based beverage fermented by kefir grains have already been designed [160]. The combination of apple juice and kefir grains resulted in a beverage with high total phenolic content and antioxidant activity.

## 6. Conclusions

This review emphasized the microbial ecosystem of musts and showed how mastering the quality and the safety of cider production is reliant on a better understanding of the mechanisms of LAB and yeast metabolism involved in the transformation of precursors into potent flavor components. The present review further paved the way for the optimization of the industrial scale-up for artisanal cider production using the integrated metabolomics and molecular phylogeny approaches to identify and select strains of LAB, particularly *O. oeni*, to improve the flavor/aroma profiles of ciders. Indeed, although considerable efforts have been made in recent decades to optimize and improve the production of cider, cider remains a product with a great variability related in particular to the notion of ‘terroir’ that can be defined as a homogeneous territory from a soil and climate point of view. Therefore, pedoclimate factors together with indigenous microorganisms may significantly influence the quality and typicity of the cider produced in a specific location. The apple benefits from a good and healthy image that could be combined with new microbial characteristics with a special focus on LAB. Specific research on microbiomes using ‘omics’ tools will give rapid insights into the potential of strains associated with these products. For these reasons, studies on apple fermentation beverages comprise a promising field of research with great potential for available new, healthy and pleasant products on the market.

## Figures and Tables

**Table 1:** Bacterial diversity found in apple juice-related products.

Family	Origin	Genus/Species	References
*Lactobacillaceae*	Apple flower Fresh-cut apple Cider	*Lactobacillus brevis*	[24,33,34,35,36]
		*Lactobacillus (para)collinoides*	
		*Lactobacillus casei*	
		*Lactobacillus diolivorans*	
		*Lactobacillus hilgardii*	
		*Lactobacillus sicerae*	
		*Lactobacillus suebicus*	
		*Pediococcus ethanolidurans*	
		*Pediococcus parvulus*	
*Leuconostocaceae*	Cider	*Oenococcus oeni*	[37,38]
		*Leuconostoc mesenteroides*	
*Acetobacteraceae*	Apple flower Apple cider vinegar	*Acetobacter* sp.	[17,32]
		*Komagataeibacter* sp.	
		*Gluconobacter* sp.	
*Sporolactobacilliaceae*	Cider	*Sporolactobacillus* sp.	[39]
*Sphingomonadaceae*	Cider	*Zymomonas mobilis*	[40,41]
*Enterobacteriaceae*	Apple surface Apple flower	Coliforms *Enterobacteriaceae* ^a^	[24,27,32]

^a^ Genus unspecified.

**Table 2:** Potential bacterial species producers of biogenic amines in Spanish and French ciders.

Biogenic Amine	Producer	References
Histamine	*Lactobacillus paracollinoides*	[35,86,94]
	*Lactobacillus hilgardii*	
	*Lactobacillus diolivorans*	
	*Lactobacillus collinoides*	
	*Oenococcus oeni*	
Putrescine	*Lactobacillus collinoides*	[39,89]
	*Oenococcus oeni*	
	*Lactobacillus brevis*	
	*Lactobacillus mali*	
	*Leuconostoc mesenteroides*	
	*Pediococcus parvulus*	
	*Lactobacillus paracollinoides*	
Tyramine	*Sporolactobacillus* sp.	[35,86,93]
	*Lactobacillus brevis*	
	*Lactobacillus diolivorans*	
	*Oenococcus oeni*	
	*Pediococcus parvulus*	

## References

[1] Lea A.G.H., Lea A.G.H., Piggott J.R. (1995). Cidermaking. Fermented Beverage Production.

[2] Nogueira A., Wosiacki G., Hui Y.H. (2012). Apple Cider Fermentation. Handbook of Plant-Based Fermented Food and Beverage Technology.

[3] Jarvis B., Caballero B. (2003). CIDER (CYDER; HARD CIDER): The Product and its Manufacture. Encyclopedia of Food Sciences and Nutrition.

[4] AICV—Publications. http://www.aicv.org/pages/aicv/publications.html.

[5] Richards A., Morgan J. (1993). The Book of Apples.

[6] Watson B. (2013). Cider Styles and Traditions. Cider, Hard and Sweet: History, Traditions, and Making Your Own.

[7] Downing D.L. (1989). Apple cider. Processed Apple Products.

[8] National Apple Museum. http://www.nationalapplemuseum.com/appleciderandmore.html.

[9] Coton E., Coton M., Guichard H. (2016). Cider (Cyder; Hard Cider): The Product and Its Manufacture. Encyclopedia of Food and Health.

[10] Boulongne I. La Vie Paysanne Autrefois. http://viepaysanneautrefois.free.fr/.

[11] L’histoire du Cidre en France. http://www.lalittorale.fr/lhistoire-du-cidre-en-france/.

[12] Jarvis B., Lea A.G.H. (2000). Sulphite binding in ciders. Int. J. Food Sci. Technol..

[13] Sánchez A., Rodríguez R., Coton M., Coton E., Herrero M., García L.A., Díaz M. (2010). Population dynamics of lactic acid bacteria during spontaneous malolactic fermentation in industrial cider. Food Res. Int..

[14] Beech F.W., Rose A.H., Harrison J.S. (1993). 5—Yeasts in Cider-Making. The Yeasts.

[15] Handelsman J. (2009). Metagenetics: Spending our inheritance on the future. Microb. Biotechnol..

[16] Tringe S.G., Hugenholtz P. (2008). A renaissance for the pioneering 16S rRNA gene. Curr. Opin. Microbiol..

[17] Trček J., Mahnič A., Rupnik M. (2016). Diversity of the microbiota involved in wine and organic apple cider submerged vinegar production as revealed by DHPLC analysis and next-generation sequencing. Int. J. Food Microbiol..

[18] David V., Terrat S., Herzine K., Claisse O., Rousseaux S., Tourdot-Maréchal R., Masneuf-Pomarede I., Ranjard L., Alexandre H. (2014). High-throughput sequencing of amplicons for monitoring yeast biodiversity in must and during alcoholic fermentation. J. Ind. Microbiol. Biotechnol..

[19] Makarova K., Slesarev A., Wolf Y., Sorokin A., Mirkin B., Koonin E., Pavlov A., Pavlova N., Karamychev V., Polouchine N. (2006). Comparative genomics of the lactic acid bacteria. Proc. Natl. Acad. Sci. USA.

[20] Sun Z., Harris H.M.B., McCann A., Guo C., Argimón S., Zhang W., Yang X., Jeffery I.B., Cooney J.C., Kagawa T.F. (2015). Expanding the biotechnology potential of lactobacilli through comparative genomics of 213 strains and associated genera. Nat. Commun..

[21] Herrera C.M., de Vega C., Canto A., Pozo M.I. (2009). Yeasts in floral nectar: A quantitative survey. Ann. Bot..

[22] Pozo M.I., Herrera C.M., Bazaga P. (2011). Species richness of yeast communities in floral nectar of southern Spanish plants. Microb. Ecol..

[23] Pusey P.L., Stockwell V.O., Mazzola M. (2009). Epiphytic bacteria and yeasts on apple blossoms and their potential as antagonists of *Erwinia amylovora*. Phytopathology.

[24] Graça A., Santo D., Esteves E., Nunes C., Abadias M., Quintas C. (2015). Evaluation of microbial quality and yeast diversity in fresh-cut apple. Food Microbiol..

[25] Batzer J.C., Sisson A.J., Harrington T.C., Mayfield D.A., Gleason M.L. (2012). Temporal patterns in appearance of sooty blotch and flyspeck fungi on apples. Microb. Ecol..

[26] Ismail S.I., Batzer J.C., Harrington T.C., Crous P.W., Lavrov D.V., Li H., Gleason M.L. (2016). Ancestral state reconstruction infers phytopathogenic origins of sooty blotch and flyspeck fungi on apple. Mycologia.

[27] Alonso S., Laca A., Rendueles M., Mayo B., Díaz M. (2015). Cider apple native microbiota characterization by PCR-DGGE. J. Inst. Brew..

[28] Morrissey W.F., Davenport B., Querol A., Dobson A.D. (2004). The role of indigenous yeasts in traditional Irish cider fermentations. J. Appl. Microbiol..

[29] Coton E., Coton M., Levert D., Casaregola S., Sohier D. (2006). Yeast ecology in French cider and black olive natural fermentations. Int. J. Food. Microbiol..

[30] Suarez Valles B., Bedrinana R.P., Tascon N.F., Simon A.Q., Madrera R.R. (2007). Yeast species associated with the spontaneous fermentation of cider. Food Microbiol..

[31] Pando Bedrinana R., Querol Simon A., Suarez Valles B. (2010). Genetic and phenotypic diversity of autochthonous cider yeasts in a cellar from Asturias. Food Microbiol..

[32] Shade A., McManus P.S., Handelsman J. (2013). Unexpected Diversity during Community Succession in the Apple Flower Microbiome. mBio.

[33] Sánchez A., Coton M., Coton E., Herrero M., García L.A., Díaz M. (2012). Prevalent lactic acid bacteria in cider cellars and efficiency of *Oenococcus oeni* strains. Food Microbiol..

[34] Claisse O., Lonvaud-Funel A. (2001). Détection de bactéries lactiques produisant du 3-hydroxypropionaldéhyde (précurseur d’acroléine) à partir du glycérol par tests moléculaires. Le Lait.

[35] Puertas A.I., Arahal D.R., Ibarburu I., Elizaquível P., Aznar R., Dueñas M.T. (2014). *Lactobacillus sicerae* sp. nov., a lactic acid bacterium isolated from Spanish natural cider. Int. J. Syst. Evol. Microbiol..

[36] Garai G., Dueñas M.T., Irastorza A., Moreno-Arribas M.V. (2007). Biogenic amine production by lactic acid bacteria isolated from cider. Lett. Appl. Microbiol..

[37] Salih A.G., Drilleau J.F., Cavin F.F., Divies C., Bourgeois C.M. (1988). A Survey of Microbiological Aspects of Cider Making. J. Inst. Brew..

[38] El Khoury M., Campbell-Sills H., Salin F., Guichoux E., Claisse O., Lucas P.M. (2017). Biogeography of *Oenococcus oeni* reveals distinctive but nonspecific populations in wine-producing regions. Appl. Environ. Microbiol..

[39] Coton M., Romano A., Spano G., Ziegler K., Vetrana C., Desmarais C., Lonvaud-Funel A., Lucas P., Coton E. (2010). Occurrence of biogenic amine-forming lactic acid bacteria in wine and cider. Food Microbiol..

[40] Swings J., De Ley J. (1977). The biology of *Zymomonas*. Bacteriol. Rev..

[41] Bauduin R., Le Quere J.M., Coton E., Primault J. (2006). Factors leading to the expression of ‘framboisé’ in French ciders. LWT Food Sci. Technol..

[42] Keller S.E., Chirtel S.J., Merker R.I., Taylor K.T., Tan H.L., Miller A.J. (2004). Influence of fruit variety, harvest technique, quality sorting, and storage on the native microflora of unpasteurized apple cider. J. Food Prot..

[43] Mills D.A., Rawsthorne H., Parker C., Tamir D., Makarova K. (2005). Genomic analysis of *Oenococcus oeni* PSU-1 and its relevance to winemaking. FEMS Microbiol. Rev..

[44] Bridier J., Claisse O., Coton M., Coton E., Lonvaud-Funel A. (2010). Evidence of distinct populations and specific subpopulations within the species *Oenococcus oeni*. Appl. Environ. Microbiol..

[45] Campbell-Sills H., El Khoury M., Favier M., Romano A., Biasioli F., Spano G., Sherman D.J., Bouchez O., Coton E., Coton M. (2015). Phylogenomic analysis of *Oenococcus oeni* reveals specific domestication of strains to cider and wines. Genome Biol. Evol..

[46] Sternes P.R., Borneman A.R. (2016). Consensus pan-genome assembly of the specialised wine bacterium *Oenococcus oeni*. BMC Genom..

[47] Pérez-Ramos A., Mohedano M.L., Puertas A., Lamontanara A., Orru L., Spano G., Capozzi V., Dueñas M.T., López P. (2016). Draft genome sequence of *Pediococcus parvulus* 2.6, a probiotic β-glucan producer strain. Genome Announc..

[48] Puertas A.I., Capozzi V., Llamas M.G., López P., Lamontanara A., Orrù L., Russo P., Spano G., Dueñas M.T. (2016). Draft genome sequence of *Lactobacillus collinoides* CUPV237, an exopolysaccharide and riboflavin producer isolated from cider. Genome Announc..

[49] Ottesen A.R., White J.R., Skaltsas D.N., Newell M.J., Walsh C.S. (2009). Impact of organic and conventional management on the phyllosphere microbial ecology of an apple crop. J. Food Prot..

[50] Yashiro E., Spear R.N., McManus P.S. (2011). Culture-dependent and culture-independent assessment of bacteria in the apple phyllosphere. J. Appl. Microbiol..

[51] Granado J., Thurig B., Kieffer E., Petrini L., Fliessbach A., Tamm L., Weibel F.P., Wyss G.S. (2008). Culturable fungi of stored ‘golden delicious’ apple fruits: A one-season comparison study of organic and integrated production systems in Switzerland. Microb. Ecol..

[52] Fleet G.H. (2003). Yeast interactions and wine flavour. Int. J. Food Microbiol..

[53] Azhu Valappil Z., Fan X., Zhang H.Q., Rouseff R.L. (2009). Impact of thermal and nonthermal processing technologies on unfermented apple cider aroma volatiles. J. Agric. Food Chem..

[54] Lilly M., Lambrechts M.G., Pretorius I.S. (2000). Effect of increased yeast alcohol acetyltransferase activity on flavor profiles of wine and distillates. Appl. Environ. Microbiol..

[55] Xu Y., Zhao G.A., Wang L.P. (2006). Controlled formation of volatile components in cider making using a combination of *Saccharomyces cerevisiae* and *Hanseniaspora valbyensis* yeast species. J. Ind. Microbiol. Biotechnol..

[56] Sumby K.M., Grbin P.R., Jiranek V. (2010). Microbial modulation of aromatic esters in wine: Current knowledge and future prospects. Food Chem..

[57] Mangas J.J., Cabranes C., Moreno J., Gomis D.B. (1994). Influence of Cider-Making Technology on Cider Taste. LWT Food Sci. Technol..

[58] Herrero M. (2011). Influence des Fermentations Alcoolique et Malolactique sur la Composition Chimique des Cidres À Distiller en Cours D’élaboaration.

[59] Rapp A., Mandery H. (1987). New progess in wine and wine research. Experientia.

[60] Rous C.V., Snow R., Kunkee R.E. (1983). Reduction of Higher Alcohols by Fermentation with a Leucine-Auxotrophic Mutant of Wine Yeast. J. Inst. Brew..

[61] Jepsen O.M. (1978). The sensory and analitical evaluation of apple juice volatiles. Int. Fed. Fruit Juice Prod..

[62] Ye M., Yue T., Yuan Y. (2014). Evolution of polyphenols and organic acids during the fermentation of apple cider. J. Sci. Food Agric..

[63] Lea A.G.H., Arnold G.M. (1978). The Phenolics of Cider: Bitterness and Astringency. J. Sci. Food Agric..

[64] Symoneaux R., Baron A., Marnet N., Bauduin R., Chollet S. (2014). Impact of apple procyanidins on sensory perception in model cider (part 1): Polymerisation degree and concentration. LWT Food Sci. Technol..

[65] Symoneaux R., Chollet S., Bauduin R., Le Quéré J.M., Baron A. (2014). Impact of apple procyanidins on sensory perception in model cider (part 2): Degree of polymerization and interactions with the matrix components. LWT Food Sci. Technol..

[66] Vanbeneden N., Van Roey T., Willems F., Delvaux F., Delvaux F.R. (2008). Release of phenolic flavour precursors during wort production: Influence of process parameters and grist composition on ferulic acid release during brewing. Food Chem..

[67] Stratford M., James S.A., Boekhout T., Robert V. (2003). 12-Non-alcoholic beverages and yeasts. Yeasts in Food.

[68] Smith B.D., Divol B. (2016). *Brettanomyces bruxellensis*, a survivalist prepared for the wine apocalypse and other beverages. Food Microbiol..

[69] Buron N., Coton M., Legendre P., Ledauphin J., Kientz-Bouchart V., Guichard H., Barillier D., Coton E. (2012). Implications of Lactobacillus collinoides and Brettanomyces/Dekkera anomala in phenolic off-flavour defects of ciders. Int. J. Food Microbiol..

[70] Wedral D., Shewfelt R., Frank J. (2010). The challenge of *Brettanomyces* in wine. LWT Food Sci. Technol..

[71] Mehlomakulu N.N., Setati M.E., Divol B. (2014). Characterization of novel killer toxins secreted by wine-related non-*Saccharomyces* yeasts and their action on *Brettanomyces* spp.. Int. J. Food Microbiol..

[72] Quirós C., Herrero M., García L.A., Díaz M. (2012). Effects of SO2 on lactic acid bacteria physiology when used as a preservative compound in malolactic fermentation. J. Inst. Brew..

[73] Lonvaud-Funel A. (1999). Lactic acid bacteria in the quality improvement and depreciation of wine. Antonie Leeuwenhoek.

[74] Henick-Kling T. (1995). Control of malolactic fermentation in wine: Energetics, flavour modification and methods of starter culture preparation. J. Appl. Bacteriol..

[75] Osborne J.P., Mira de Orduña R., Pilone G.J., Liu S.Q. (2000). Acetaldehyde metabolism by wine lactic acid bacteria. FEMS Microbiol. Lett..

[76] Ugliano M., Moio L. (2005). Changes in the concentration of yeast-derived volatile compounds of red wine during malolactic fermentation with four commercial starter cultures of Oenococcus oeni. J. Agric. Food Chem..

[77] Swiegers J.H., Bartowsky E.J., Henschke P.A., Pretorius I.S. (2005). Yeast and bacterial modulation of wine aroma and flavour. Aust. J. Grape Wine Res..

[78] Maicas S., Gil J.V., Pardo I., Ferrer S. (1999). Improvement of volatile composition of wines by controlled addition of malolactic bacteria. Food Res. Int..

[79] Bartowsky E.J., Henschke P.A. (2004). The ‘buttery’ attribute of wine—diacetyl—desirability, spoilage and beyond. Int. J. Food Microbiol..

[80] Sauvageot N., Muller C., Hartke A., Auffray Y., Laplace J.-M. (2002). Characterisation of the diol dehydratase *pdu* operon of *Lactobacillus collinoides*. FEMS Microbiol. Lett..

[81] Sauvageot N., Pichereau V., Louarme L., Hartke A., Auffray Y., Laplace J.-M. (2002). Purification, characterization and subunits identification of the diol dehydratase of *Lactobacillus collinoides*. Eur. J. Biochem..

[82] Garai-Ibabe G., Ibarburu I., Berregi I., Claisse O., Lonvaud-Funel A., Irastorza A., Dueñas M.T. (2008). Glycerol metabolism and bitterness producing lactic acid bacteria in cidermaking. Int. J. Food Microbiol..

[83] Ledauphin J., Lefrancois A., Marquet N., Beljean-Leymarie M., Barillier D. (2006). Development of an accurate and sensitive gas chromatographic method for the determination of acrolein content in Calvados and cider. LWT Food Sci. Technol..

[84] Carr J.G., Davies P.A. (1972). The Ecology and Classification of Strains of *Lactobacillus collinoides* nov. spec.: A Bacterium Commonly Found in Fermenting Apple Juice. J. Appl. Bacteriol..

[85] Sauvageot N., Gouffi K., Laplace J.M., Auffray Y. (2000). Glycerol metabolism in *Lactobacillus collinoides*: Production of 3-hydroxypropionaldehyde, a precursor of acrolein. Int. J. Food Microbiol..

[86] Coton M., Laplace J.M., Coton E. (2005). *Zymomonas mobilis* subspecies identification by amplified ribosomal DNA restriction analysis. Lett. Appl. Microbiol..

[87] Jarvis B., Batt C.A., Tortorello M.L. (2014). Cider (Cyder; Hard Cider). Encyclopedia of Food Microbiology.

[88] Ladero V., Cruz Martin M., Fernandez M., Alvarez M.A. (2010). Toxicological Effects of Dietary Biogenic Amines. Curr. Nutr. Food Sci..

[89] Ladero V., Coton M., Fernández M., Buron N., Martín M.C., Guichard H., Coton E., Alvarez M.A. (2011). Biogenic amines content in Spanish and French natural ciders: Application of qPCR for quantitative detection of biogenic amine-producers. Food Microbiol..

[90] Izquierdo-Pulido M., Albalá-Hurtado S., Mariné-Font A., Vidal-Carou M.C. (1996). Biogenic amines in Spanish beers: Differences among breweries. Z. Lebensm. Unters. Forsch..

[91] Marcobal A., Martín-Alvarez P.J., Polo M.C., Muñoz R., Moreno-Arribas M.V. (2006). Formation of biogenic amines throughout the industrial manufacture of red wine. J. Food Prot..

[92] Vidal-Carou M.C., Ambatlle-Espunyes A., Ulla-Ulla M.C., Mariné-Font A. (1990). Histamine and Tyramine in Spanish Wines: Their Formation During the Winemaking Process. Am. J. Enol. Vitic..

[93] Martín-Álvarez P.J., Marcobal Á., Polo C., Moreno-Arribas M.V. (2006). Influence of technological practices on biogenic amine contents in red wines. Eur. Food Res. Technol..

[94] Alvarez M.A., Moreno-Arribas M.V. (2014). The problem of biogenic amines in fermented foods and the use of potential biogenic amine-degrading microorganisms as a solution. Trends Food Sci. Technol..

[95] Garai-Ibabe G., Irastorza A., Dueñas M.T., Martín-Álvarez P.J., Moreno-Arribas V.M. (2013). Evolution of amino acids and biogenic amines in natural ciders as a function of the year and the manufacture steps. Int. J. Food Sci. Technol..

[96] Del Campo G., Lavado I., Dueñas M., Irastoza M. (2000). Histamine production by some lactic acid bacteria isolated from ciders. Food Sci. Technol. Int..

[97] Callejón S., Sendra R., Ferrer S., Pardo I. (2014). Identification of a novel enzymatic activity from lactic acid bacteria able to degrade biogenic amines in wine. Appl. Microbiol. Biotechnol..

[98] Binder E.M., Tan L.M., Chin L.J., Handl J., Richard J. (2007). Worldwide occurrence of mycotoxins in commodities, feeds and feed ingredients. Anim. Feed Sci. Technol..

[99] Moake M.M., Padilla-Zakour O.I., Worobo R.W. (2005). Comprehensive Review of Patulin Control Methods in Foods. Compr. Rev. Food Sci. Food Saf..

[100] McKinley E.R., Carlton W.W., Shanna R.P., Salunkhe D.K. (1991). Patulin. Mycotoxins and Phytoalexins.

[101] Postupolski J., Rybińska K., Kurpińska-Jaworska J., Ledzion E., Szczesna M., Karłowski K. (2003). European Union legislation related to patulin. Rocz. Panstw. Zakl. Hig..

[102] Harris K.L., Bobe G., Bourquin L.D. (2009). Patulin surveillance in apple cider and juice marketed in Michigan. J. Food Prot..

[103] Tannous J., Atoui A., El Khoury A., Francis Z., Oswald I.P., Puel O., Lteif R. (2016). A study on the physicochemical parameters for *Penicillium expansum* growth and patulin production: Effect of temperature, pH, and water activity. Food Sci. Nutr..

[104] Fernández-Cruz M.L., Mansilla M.L., Tadeo J.L. (2010). Mycotoxins in fruits and their processed products: Analysis, occurrence and health implications. J. Adv. Res..

[105] Pollution du Fruit et Patuline−IFPC—Cidre, Pomme, Pommier. http://www.ifpc.eu/bibliographie/recolte-qualite-des-fruits/qualite-du-fruit.html.

[106] Mihajlovic B., Dixon B., Couture H., Farber J. (2013). Qualitative Microbiological Risk Assessment of Unpasteurized Fruit Juice and Cider. Int. Food Risk Anal. J..

[107] Merwin I.A., Valois S., Padilla-Zakour O.I., Janick J. (2007). Cider Apples and Cider-Making Techniques in Europe and North America. Horticultural Reviews.

[108] Garcia L., Henderson J., Fabri M., Oke M. (2006). Potential sources of microbial contamination in unpasteurized apple cider. J. Food Prot..

[109] Kniel K.E., Sumner S.S., Lindsay D.S., Hackney C.R., Pierson M.D., Zajac A.M., Golden D.A., Fayer R. (2003). Effect of organic acids and hydrogen peroxide on *Cryptosporidium parvum* viability in fruit juices. J. Food Prot..

[110] Bartowsky E.J. (2005). *Oenococcus oeni* and malolactic fermentation-moving into the molecular arena. Aust. J. Grape Wine Res..

[111] Magalhães F., Krogerus K., Vidgren V., Sandell M., Gibson B. (2017). Improved cider fermentation performance and quality with newly generated *Saccharomyces cerevisiae* × *Saccharomyces eubayanus* hybrids. J. Ind. Microbiol. Biotechnol..

[112] Betteridge A., Grbin P., Jiranek V. (2015). Improving *Oenococcus oeni* to overcome challenges of wine malolactic fermentation. Trends Biotechnol..

[113] Schümann C., Michlmayr H., Eder R., del Hierro A.M., Kulbe K.D., Mathiesen G., Nguyen T.-H. (2012). Heterologous expression of *Oenococcus oeni* malolactic enzyme in *Lactobacillus plantarum* for improved malolactic fermentation. AMB Express.

[114] Du Toit M., Engelbrecht L., Lerm E., Krieger-Weber S. (2011). *Lactobacillus*: The next generation of malolactic fermentation starter cultures—An overview. Food Bioprocess Technol..

[115] Suárez Valles B., Pando Bedriñana R., Lastra Queipo A., Mangas Alonso J.J. (2008). Screening of cider yeasts for sparkling cider production (Champenoise method). Food Microbiol..

[116] De Arruda Moura Pietrowski G., dos Santos C.M.E., Sauer E., Wosiacki G., Nogueira A. (2012). Influence of fermentation with *Hanseniaspora* sp. yeast on the volatile profile of fermented apple. J. Agric. Food Chem..

[117] Kot W., Neve H., Heller K.J., Vogensen F.K. (2014). Bacteriophages of *Leuconostoc*, *Oenococcus*, and *Weissella*. Front. Microbiol..

[118] Costantini A., Doria F., Saiz J.-C., Garcia-Moruno E. (2017). Phage-host interactions analysis of newly characterized Oenococcus oeni bacteriophages: Implications for malolactic fermentation in wine. Int. J. Food Microbiol..

[119] Endersen L., O’Mahony J., Hill C., Ross R.P., McAuliffe O., Coffey A. (2014). Phage therapy in the food industry. Annu. Rev. Food Sci. Technol..

[120] Oliveira M., Viñas I., Colàs P., Anguera M., Usall J., Abadias M. (2014). Effectiveness of a bacteriophage in reducing *Listeria monocytogenes* on fresh-cut fruits and fruit juices. Food Microbiol..

[121] Aneja K.R., Dhiman R., Aggarwal N.K., Aneja A. (2014). Emerging preservation techniques for controlling spoilage and pathogenic microorganisms in fruit juices. Int. J. Microbiol..

[122] Ye M., Yue T., Yuan Y. (2014). Effects of sequential mixed cultures of *Wickerhamomyces anomalus* and *Saccharomyces cerevisiae* on apple cider fermentation. FEMS Yeast Res..

[123] Priya P., Munishamanna K.B. (2013). Microbial fermentation of blended tomato juice by yeast and lactic acid bacteria for nutritional improvement. Environ. Ecol..

[124] Peng B., Li F., Cui L., Guo Y. (2015). Effects of fermentation temperature on key aroma compounds and sensory properties of apple wine. J. Food Sci..

[125] Herrero M., García L.A., Díaz M. (2006). Volatile compounds in cider: Inoculation time and fermentation temperature effects. J. Inst. Brew..

[126] Satora P., Tarko T., Duda-Chodak A., Sroka P., Tuszyński T., Czepielik M. (2009). Influence of prefermentative treatments and fermentation on the antioxidant and volatile profiles of apple wines. J. Agric. Food Chem..

[127] Zhang D., Lovitt R.W. (2006). Strategies for enhanced malolactic fermentation in wine and cider maturation. J. Chem. Technol. Biotechnol..

[128] Devi Avaiyarasi N., David Ravindran A., Venkatesh P., Arul V. (2016). In vitro selection, characterization and cytotoxic effect of bacteriocin of *Lactobacillus sakei* GM3 isolated from goat milk. Food Control.

[129] Wen L.S., Philip K., Ajam N. (2016). Purification, characterization and mode of action of plantaricin K25 produced by *Lactobacillus plantarum*. Food Control.

[130] Hu Y., Liu X., Shan C., Xia X., Wang Y., Dong M., Zhou J. (2017). Novel bacteriocin produced by *Lactobacillus alimentarius* FM-MM4 from a traditional Chinese fermented meat Nanx Wudl: Purification, identification and antimicrobial characteristics. Food Control.

[131] Laplace J.M., Sauvageot N., Hartke A., Auffray Y. (1999). Characterization of *Lactobacillus collinoides* response to heat, acid and ethanol treatments. Appl. Microbiol. Biotechnol..

[132] Gálvez A., Maqueda M., Valdivia E., Quesada A., Montoya E. (1986). Characterization and partial purification of a broad spectrum antibiotic AS-48 produced by *Streptococcus faecalis*. Can. J. Microbiol..

[133] Martínez-Viedma P., Abriouel H., Omar N.B., Valdivia E., López R.L., Gálvez A. (2008). Inactivation of exopolysaccharide and 3-hydroxypropionaldehyde-producing lactic acid bacteria in apple juice and apple cider by enterocin AS-48. Food Chem. Toxicol. Int. J. Public Br. Ind. Biol. Res. Assoc..

[134] Dueñas M., Irastorza A., Fernández K., Bilbao A. (1995). Heterofermentative Lactobacilli causing ropiness in Basque country ciders. J. Food Prot..

[135] Ibarburu I., Puertas A.I., Berregi I., Rodríguez-Carvajal M.A., Prieto A., Dueñas M.T. (2015). Production and partial characterization of exopolysaccharides produced by two *Lactobacillus suebicus* strains isolated from cider. Int. J. Food Microbiol..

[136] Korakli M., Gänzle M.G., Vogel R.F. (2002). Metabolism by bifidobacteria and lactic acid bacteria of polysaccharides from wheat and rye, and exopolysaccharides produced by *Lactobacillus sanfranciscensis*. J. Appl. Microbiol..

[137] Dueñas-Chasco M.T., Rodríguez-Carvajal M.A., Tejero Mateo P., Franco-Rodríguez G., Espartero J.L., Irastorza-Iribas A., Gil-Serrano A.M. (1997). Structural analysis of the exopolysaccharide produced by *Pediococcus damnosus* 2.6. Carbohydr. Res..

[138] Dueñas-Chasco M.T., Rodríguez-Carvajal M.A., Tejero-Mateo P., Espartero J.L., Irastorza-Iribas A., Gil-Serrano A.M. (1998). Structural analysis of the exopolysaccharides produced by *Lactobacillus* spp. G-77. Carbohydr. Res..

[139] Ibarburu I., Soria-Díaz M.E., Rodríguez-Carvajal M.A., Velasco S.E., Tejero-Mateo P., Gil-Serrano A.M., Irastorza A., Dueñas M.T. (2007). Growth and exopolysaccharide (EPS) production by *Oenococcus oeni* I4 and structural characterization of their EPSs. J. Appl. Microbiol..

[140] Larpin S., Sauvageot N., Pichereau V., Laplace J.-M., Auffray Y. (2002). Biosynthesis of exopolysaccharide by a *Bacillus licheniformis* strain isolated from ropy cider. Int. J. Food Microbiol..

[141] Grande M.J., Lucas R., Abriouel H., Valdivia E., Ben Omar N., Maqueda M., Martínez-Cañamero M., Gálvez A. (2006). Inhibition of *Bacillus licheniformis* LMG 19409 from ropy cider by enterocin AS-48. J. Appl. Microbiol..

[142] Hassan Y.I., Zhou T., Bullerman L.B. (2016). Sourdough lactic acid bacteria as antifungal and mycotoxin-controlling agents. Rev. Agaroquim. Tecnol. Aliment..

[143] Ahmad Rather I., Seo B.J., Rejish Kumar V.J., Choi U.-H., Choi K.-H., Lim J.H., Park Y.-H. (2013). Isolation and characterization of a proteinaceous antifungal compound from *Lactobacillus plantarum* YML007 and its application as a food preservative. Lett. Appl. Microbiol..

[144] Zoghi A., Khosravi-Darani K., Sohrabvandi S., Attar H., Alavi S.A. (2016). Effect of probiotics on patulin removal from synbiotic apple juice. J. Sci. Food Agric..

[145] Juodeikiene G., Basinskiene L., Bartkiene E., Matusevicius P. (2012). Mycotoxin Decontamination Aspects in Food, Feed and Renewables Using Fermentation Processes. Structure and Function of Food Engineering.

[146] Prado F.C., Parada J.L., Pandey A., Soccol C.R. (2008). Trends in non-dairy probiotic beverages. Food Res. Int..

[147] Kandylis P., Pissaridi K., Bekatorou A., Kanellaki M., Koutinas A.A. (2016). Dairy and non-dairy probiotic beverages. Curr. Opin. Food Sci..

[148] Marsh A.J., Hill C., Ross R.P., Cotter P.D. (2014). Fermented beverages with health-promoting potential: Past and future perspectives. Trends Food Sci. Technol..

[149] Konowalchuk J., Speirs J.I. (1978). Antiviral effect of apple beverages. Appl. Environ. Microbiol..

[150] Gawkowski D., Chikindas M.L. (2013). Non-dairy probiotic beverages: The next step into human health. Benef. Microbes.

[151] De Souza Neves Ellendersen L., Granato D., Bigetti Guergoletto K., Wosiacki G. (2012). Development and sensory profile of a probiotic beverage from apple fermented with *Lactobacillus casei*. Eng. Life Sci..

[152] Sornplang P., Piyadeatsoontorn S. (2016). Probiotic isolates from unconventional sources: A review. J. Anim. Sci. Technol..

[153] Upadrasta A., O’Sullivan L., O’Sullivan O., Sexton N., Lawlor P.G., Hill C., Fitzgerald G.F., Stanton C., Ross R.P. (2013). The effect of dietary supplementation with spent cider yeast on the Swine distal gut microbiome. PLoS ONE.

[154] Fernández de Palencia P., Werning M.L., Sierra-Filardi E., Dueñas M.T., Irastorza A., Corbí A.L., López P. (2009). Probiotic properties of the 2-substituted (1,3)-β-d-glucan-producing bacterium *Pediococcus parvulus* 2.6. Appl. Environ. Microbiol..

[155] Waters D.M., Mauch A., Coffey A., Arendt E.K., Zannini E. (2015). Lactic acid bacteria as a cell factory for the delivery of functional biomolecules and ingredients in cereal-based beverages: A review. Crit. Rev. Food Sci. Nutr..

[156] García-Ruiz A., González de Llano D., Esteban-Fernández A., Requena T., Bartolomé B., Moreno-Arribas M.V. (2014). Assessment of probiotic properties in lactic acid bacteria isolated from wine. Food Microbiol..

[157] Foligné B., Dewulf J., Breton J., Claisse O., Lonvaud-Funel A., Pot B. (2010). Probiotic properties of non-conventional lactic acid bacteria: Immunomodulation by *Oenococcus oeni*. Int. J. Food Microbiol..

[158] Dimopoulou M., Bardeau T., Ramonet P.-Y., Miot-Certier C., Claisse O., Doco T., Petrel M., Lucas P., Dols-Lafargue M. (2016). Exopolysaccharides produced by *Oenococcus oeni*: From genomic and phenotypic analysis to technological valorization. Food Microbiol..

[159] Zannini E., Waters D.M., Coffey A., Arendt E.K. (2016). Production, properties, and industrial food application of lactic acid bacteria-derived exopolysaccharides. Appl. Microbiol. Biotechnol..

[160] Sabokbar N., Khodaiyan F., Moosavi-Nasab M. (2015). Optimization of processing conditions to improve antioxidant activities of apple juice and whey based novel beverage fermented by kefir grains. J. Food Sci. Technol..

